# Seasonal patterns in the epidemiology of Bell's palsy in Hungary

**DOI:** 10.3389/fneur.2023.1188137

**Published:** 2023-06-20

**Authors:** Eszter Varga, Ulambayar Battamir, István Szegedi, Lilla Hudák, Nóra Kovács, Attila Csaba Nagy

**Affiliations:** ^1^Department of Neurology, Faculty of Medicine, University of Debrecen, Debrecen, Hungary; ^2^Department of Health Informatics, Faculty of Health Sciences, University of Debrecen, Debrecen, Hungary; ^3^Department of Public Health and Epidemiology, Faculty of Medicine, University of Debrecen, Debrecen, Hungary

**Keywords:** facial nerve paralysis, Bell's palsy, recurrence, seasonality, epidemiology

## Abstract

**Introduction:**

Epidemiological data on Bell's palsy are vital for elucidating disease prevalence and enhancing therapeutic options. Our objective was to explore the prevalence and possible risk factors associated with Bell's palsy recurrence in the Clinical Center of the University of Debrecen service area. Secondary data analysis was performed using hospital discharge data, including patient information and comorbidities.

**Methods:**

Data was obtained from the Clinical Center of the University of Debrecen, on Bell's palsy patients who were treated at the hospital between January 1, 2015 and December 31, 2021. Multiple logistic regression analysis was used to examine the factors associated with Bell's palsy recurrence.

**Results:**

Of the 613 patients analyzed, 5.87% had recurrent paralysis, and the median time interval between episodes was 315 days. Hypertension was significantly associated with Bell's palsy recurrence. Moreover, seasonal distribution analysis revealed that the number of Bell's palsy episodes was higher in colder seasons, with spring and winter having a significantly higher number of episodes than summer and autumn.

**Discussion:**

This study provides insights into the prevalence and associated risk factors of Bell's palsy recurrence, which could aid in its management and help reduce the long-term consequences of the disease. Further research is necessary to determine the precise mechanisms underlying these findings.

## 1. Introduction

Despite the fact that Bell's palsy is a widespread condition, the epidemiological data on the disease are scarce (1). Bell's palsy can be described as an idiopathic, rapid-onset facial nerve paralysis. It is the most prevalent cause of facial paralysis, and it is defined by the sudden development of lower motor neuron weakening in the facial nerve with no apparent explanation (2).

Infectious, immunological and ischemic processes are all implicated in the pathogenesis of Bell's palsy (3). The most likely explanation is still the viral one, which suggests that the neurotropic herpes simplex (HSV-1, HSV-2) and Varicella zoster viruses reactivated in the geniculate ganglion (4, 5). In addition, other viral infections and autoimmune disease has also been suggested as potential pathomechanisms (6).

In different groups, the yearly incidence of Bell's palsy has been found to range from 11 to 40/100,000 people (7), with a one-in-sixty chance of developing the disease in one's lifetime. In around 71% of untreated instances, the disease resolves completely (8, 9).

About 30% of patients are expected to experience long-term consequence, including incomplete eye closure, crocodile tears, oral incompetence during eating and drinking, articulation problems, muscular contracture, synkinesis, and facial discomfort. Facial palsy can negatively affect psychological wellbeing, quality of life and cause functional and aesthetic deficits (10). Patients with face paresis could experience lower social functioning, since verbal communication and the expression of emotions are impaired by loss of facial function (11). The prevalence of anxiety and depression are higher among them (12), and psychosocial dysfunctions related to facial palsy are more common in women (13).

The variables linked to the occurrence of Bell's palsy, such as age, gender, season, pregnancy, and diabetes mellitus, have been subjected to much debate (9, 10, 14).

The aim of this research was to explore the possible influencing factors and to estimate the prevalence of the reoccurrence of Bell's palsy in the service area of the Clinical Center of University of Debrecen. Moreover, we also aimed to describe the seasonality of the disease.

## 2. Materials and methods

Based on the information from the Clinical Center of University of Debrecen, secondary data analysis was carried out using hospital discharge data (including all outpatient and inpatient medical records). Bell's palsy cases were identified by ICD-10 codes (G51.0). The data contained detailed information (age, gender, risk factors, date of admission and discharge, comorbidities, side of the paralysis) on Bell's palsy patients treated at the institution between January 1, 2015, and December 31, 2021. To identify reoccurrence of cases, we defined a minimum 90 days interval between the last and the next admission of patients (15).

The occurrence of all patients was investigated according to the months of the admission. To investigate seasonal variations in the occurrence of BP, months was classified as follows: March to May as spring, June to August as summer, September to November as autumn, and December to February as winter.

The total sample consisted of 650 patients. Patients with missing information on the side of paralysis (*n* = 37) were excluded, thus the final sample consisted of 613 patients with BP.

Text mining tools were also used to assure the diagnoses of International Classification of Diseases (ICD) codes based on free text analysis. Proportions with 95% confidence intervals were calculated for episodes in seasons. Multiple logistic regression model was used to explore the factors associated with the occurrence of Bell's palsy. Odds ratio (OR) with the corresponding 95% confidence intervals were calculated to determine the strength of association. Stata v17 (StataCorp. 2021. Stata Statistical Software: Release 17. College Station, TX: StataCorp LLC.) was used for statistical analysis; *p* < 0.05 indicated statistical significance.

The study was approved by the Regional Ethical Committee of University of Debrecen [5678-2021].

## 3. Results

Of the 613 patients 51.71% were male (*n* = 317) and 48.29% were female (*n* = 296). The age of the studied patients varied between 1 and 96 years, the mean age (±SD) was 43.02 ± 22.96 years.

**Figure 1:**
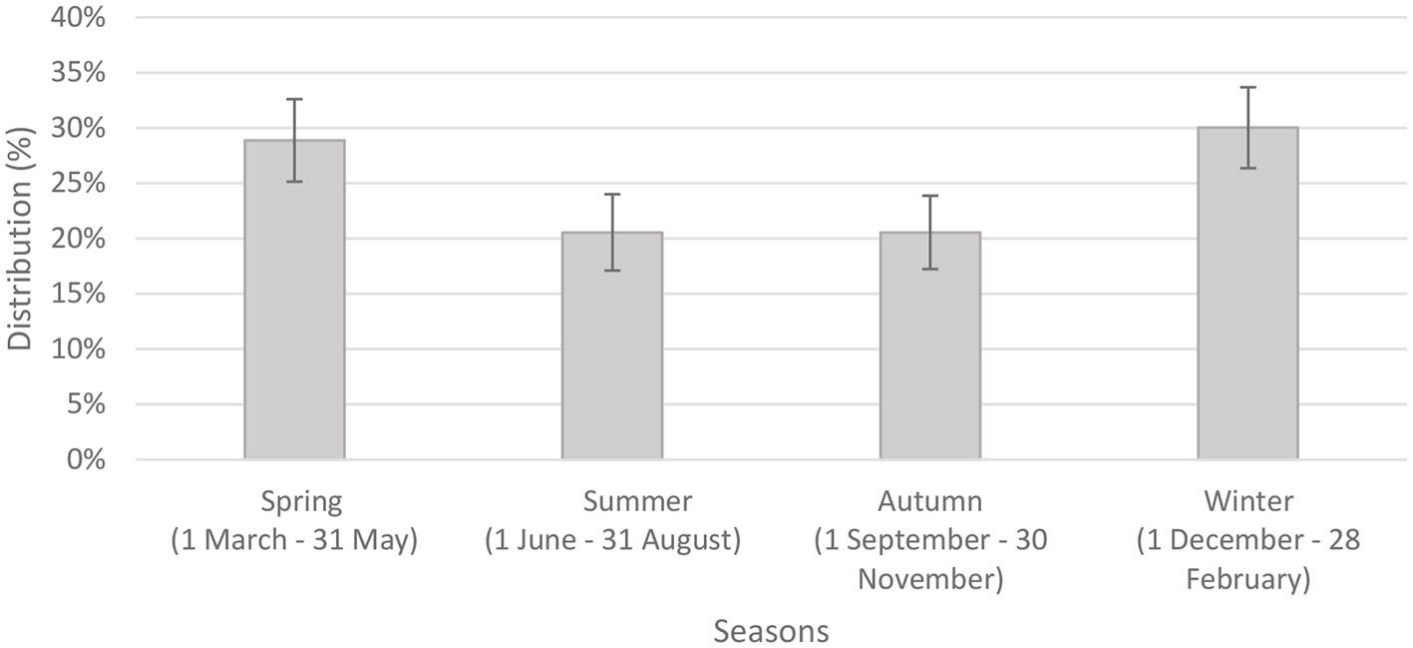
Seasonal distribution of episodes of care with 95% confidence intervals (*n* = 613).

**Figure 2:**
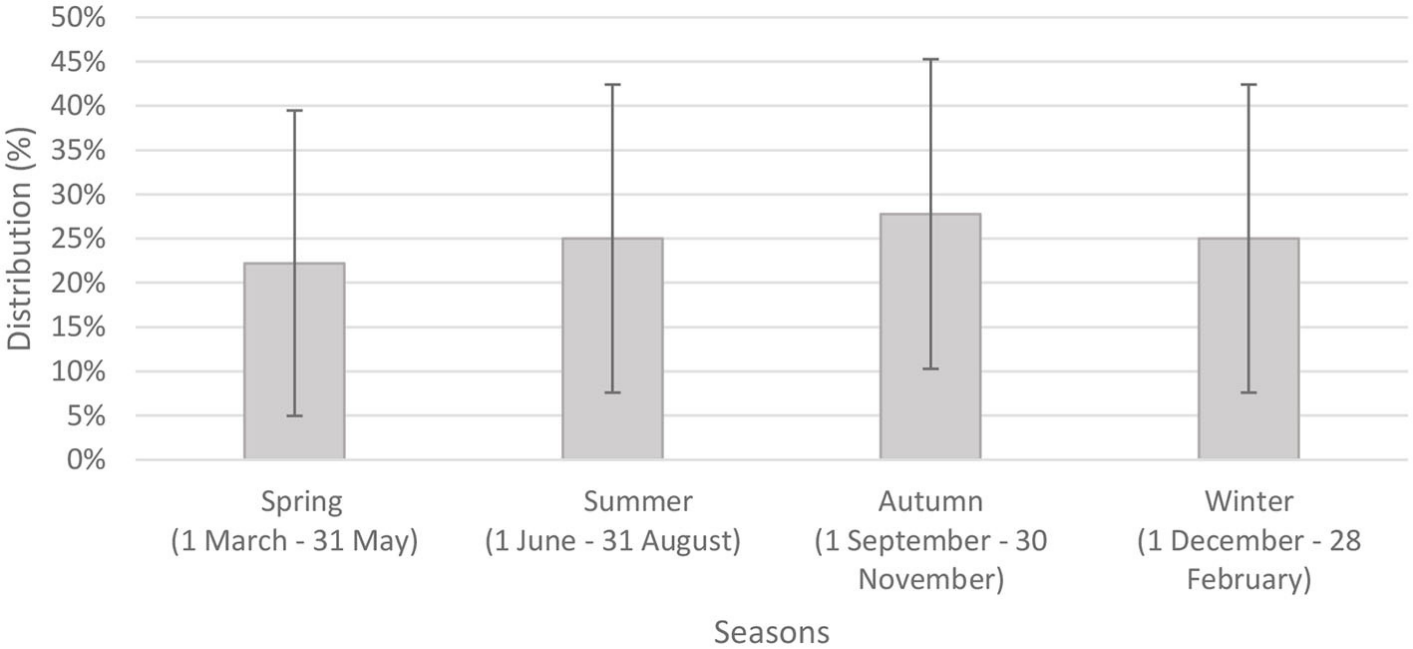
Seasonal distribution of recurrent episodes of care with 95% confidence intervals (*n* = 36).

We analyzed seasonal distribution of Bell's palsy based on episodes of care for the 613 people within the investigated period (another episode was considered separate if it occurred more than 90 days after the previous one) (15). Figure 1 shows the number of episodes in relation to seasonality. The highest number of episodes occurred in the colder seasons. The number of episodes were significantly higher in spring and winter compared to the warmer seasons. Although, there was no significant difference between seasons regarding recurrence, the highest peak was observed in autumn (Figure 2).

Thirty-six patients (5.87%) had recurrent paralysis 90 days after last admission. The median time interval between the last and next episode was 315 days.

Table 1 shows the baseline characteristics and most prevalent comorbidities occurred among patients with recurrent paresis and seasons at occurrence. Out of all the non-recurrent cases, 8.15% had non-insulin-dependent diabetes mellitus (NIDDM), 5.20% had hypertension, 1.04% had hyperlipidemia, and 0.87% had headache. On the other hand, among the recurrent cases, 11.11% had NIDDM, 16.67% had hypertension, 5.56% had hyperlipidemia, and 2.78% had headache.

**Table 1:** Baseline characteristics and prevalence of most common comorbidities and seasons for recurrence of Bell's palsy.

**Influencing factors**	**Baseline sample (*n* = 613)**	**Reccurent (*n* = 36)**	**Non-recurrent (*n* = 577)**	***p*-value**
**Demographic data**				
Age (65-X)	140 (22.84%)	7 (19.44%)	133 (23.05%)	0.617
Gender (male)	317 (51.71%)	20 (55.56%)	276 (47.83%)	0.368
**Comorbidities**				
NIDDM	51 (8.32%)	4 (11.11%)	47 (8.15%)	0.532
Hyperlipidaemia	**8 (1.31%)**	**2 (5.56%)**	**6 (1.04%)**	**0.021**
Hypertension	**36 (5.87%)**	**6 (16.67%)**	**30 (5.20%)**	**0.005**
Headache	6 (0.98%)	1 (2.78%)	5 (0.87%)	0.258
**Side of paralysis**				
Right side	215 (47.99%)	15 (46.88%)	200 (48.08%)	0.837
Left side	225 (50.22%)	16 (50.00%)	209 (50.24%)	0.837
Both sides	8 (1.79%)	1 (3.13%)	7 (1.68%)	0.837
**Season**				
Spring	177 (28.87%)	8 (22.22%)	169 (29.29%)	0.523
Summer	126 (20.55%)	9 (25.00%)	117 (20.28%)	0.524
Autumn	126 (20.55%)	10 (27.78%)	116 (20.10%)	0.525
Winter	184 (30.02%)	9 (25.00%)	175 (30.33%)	0.526

NIDDM, non-insulin-dependent diabetes mellitus.

Bold indicates significant results (*p* < 0.05).

Table 2 shows the associated factors with recurrence in a multiple logistic regression model. The results showed that the age group was not significantly associated with Bell's palsy recurrence (OR = 0.99, *p* = 0.095, 95% CI: 0.078–1.01). Gender was also not significant (OR = 1.45, *p* = 0.334, 95% CI: 0.68–3.11). However, hypertension was found to be a significant predictor of Bell's palsy recurrence (OR = 4.16, *p* = 0.007, 95% CI: 1.47–11.79). The analysis also revealed that the season was a significant predictor of Bell's palsy recurrence, where autumn compared with spring (OR = 3.16, *p* = 0.031, 95% CI: 1.11–8.97) and dyslipidemia (OR = 7.95, *p* = 0.024, 95% CI: 1.31–48.17) were significantly associated with higher odds of recurrence.

**Table 2:** Multiple logistic regression model of possible influencing factors on Bell's palsy recurrence.

**New episode**	OR^*^	***p*-value**	**95% CI**	
Age group (65-X/1–64)	0.99	0.095	0.078	1.01
Gender (female/male)	1.45	0.334	0.68	3.11
**Hypertension (presence/absence)**	**4.16**	**0.007**	**1.47**	**11.79**
Season (summer/spring)	1.98	0.222	0.66	5.90
**Season (autumn/spring)**	**3.16**	**0.031**	**1.11**	**8.97**
Season (winter/spring)	0.87	0.801	0.29	2.63
Dyslipidemia (presence/absence)	7.95	0.024	1.31	48.17

^*^The model was adjusted for each variable included in the table and for side of Bell's palsy, T2DM, headache.

OR, odds ratio; CI, confidence interval.

Bold indicates significant results (*p* < 0.05).

## 4. Discussion

In summary, age and gender were not found to be a significant factor in the model. In the present research, the mean age (±SD) was 43.02 ± 22.96 years, and 51.71% of the patients were male, which showed similar pattern to the existing literature regarding demographic composition of patients with Bell's palsy (16). According to research, the incidence of Bell's palsy is higher in adults aged 20 to 50 (9, 17–19), whereas others indicated that those aged 60 and older are more affected (20–24).

In this study, recurrent Bell's palsy was observed in 36 cases (5.87%), which was occurred mostly within 1 year after the last admission. The recurrent Bell's palsy, which can develop on either the ipsilateral or contralateral side of the first episode, is more likely to occur in the first 2 years from the onset (25). Findings of different studies are consistent with our findings, as they suggest a recurrence rate between 2.6 and 15.2% of individuals, who have already experienced an initial episode, are affected by recurrent facial palsy (26).

Our results showed that some factors were found to be associated with the recurrence such as hyperlipidemia, hypertension and seasonal factors (specifically autumn), while age, gender and NIDDM were not significant predictors.

In our study, only hypertension showed significant association with recurrence. Patients with Bell's palsy had a higher rate of arterial hypertension (27). The delicate balance of pressure systems inside the facial canal is disrupted by blood pressure variations, particularly diastolic hypertension, resulting in impaired intrafunicular circulation and, as a result, nerve injury (27).

According to the literature, diabetes mellitus, as well as hypertension, dyslipidemia, and the combination of all three comorbidities (diabetes mellitus, hypertension, and dyslipidemia) in a single patient, may affect the initial severity of blood pressure (3).

The influence of diabetes mellitus, hypertension, and dyslipidemia on the result of the facial nerve in Bell's palsy is still the subject of an ongoing debate. Some authors have established in more recent research that there is no link between diabetes mellitus, hypertension, dyslipidemia, and eventual recovery from Bell's palsy (1).

According to our results, Bell's palsy has been observed to occur more likely in spring and winter, while reoccurrence showed highest peak in autumn. Others also found that a higher incidence occurs during the cold seasons (28, 29). The association between seasons and the disease can be explained by several reasons. Kim and Park (30) presented that although low temperature and humidity are related to the onset of Bell's palsy, a marked drop in temperature (autumn) has a greater impact on the occurrence of BP than the actual low temperature (winter). Several studies have also shown associations between temperature differences and increased probability of Bell's palsy occurrence (2). Low temperature, extreme wind chill factors, and unexpected shift in atmospheric pressure have all been associated with an increased risk of Bell's palsy (30, 31). As noted earlier, the consequences of seasons and meteorological circumstances differ by climate and by techniques of data gathering and statistics, underlining the need for further well-designed studies from diverse climatic zones (30).

The specific etiology of Bell's palsy needs to be determined in order to develop targeted treatment strategies (2). Immediate treatment and referral to specialist is essential to increase the chance of full recovery (14). However, patient's self-perception of facial appearance is also necessary to evaluate the success of surgical and non-surgical interventions (32). Facial palsy has substantial impact on a individuals' life, thus measuring impairment and disability is particularly important. Several instruments have been developed to measure the impact of facial dysfunction on quality of life in facial palsy patients. The most widely used patient-reported measures are Facial Clinimetric Evaluation (FaCE) (33) scale and the Facial Disability Index (FDI) (34), which measure both physical and psychosocial function associated with facial palsy (13). Since the disease has significant psychosocial consequences, psychological and social aspects of facial palsy should be also considered during the treatment of patients in order to achieve a better quality of life.

### 4.1.Strength and limitation

The strength of this study is that the database is representative to the Eastern Hungarian population, and we were able to investigate a time period. The study has some limitations. First, although the studied population is large, extrapolation of results should be made with caution, because our study is not representative for the whole Hungarian population. We used administrative database containing hospital discharge data, in which several lifestyle and socioeconomic factors could be underestimated; therefore, these factors were not involved in this secondary data analyses.

## 5. Conclusion

Our findings highlight the importance of considering factors (gender, hypertension, season) when managing patients with Bell's palsy. Bell's palsy was more frequently observed throughout the winter and spring seasons, while the peak of reoccurrence was in autumn.

There has been a lot of debate over whether underlying comorbidities like diabetes and hypertension have a role in the recurrence of Bell's palsy.

Further research is needed to better understand the underlying mechanisms and etiology of disease to prevent recurrent Bell's palsy and improve the patients' quality of life.

## Data availability statement

The raw data supporting the conclusions of this article will be made available by the authors, without undue reservation.

## Author contributions

Study design, manuscript drafting, data collection, and analysis and interpretation: EV and AN. Critical revision of the manuscript: UB, IS, LH, and NK. All authors approval of the final version for publication.
